# Genetic Variants and Molecular Components Associated with Metabolic Dysfunctional-Associated Steatotic Liver Disease and Depression: Shared Association of ADAMTS7 and THRAP3

**DOI:** 10.3390/genes17030343

**Published:** 2026-03-19

**Authors:** Eron G. Manusov, Vincent P. Diego, Marcio Almeida, Jacob A. Galan, Kathryn Herklotz, Edwardo Abrego, Habiba Sultana, Luis Pena Marquez, Marco A. Arriaga, Marcelo Leandro, Juan Peralta, Ana C. Leandro, Tom E. Howard, Joanne E. Curran, Sandra Laston, John Blangero, Sarah Williams-Blangero

**Affiliations:** 1 Department of Human Genetics, University of Texas Rio Grande Valley, Brownsville, TX 78520, USA; 2 South Texas Diabetes and Obesity Institute, University of Texas Rio Grande Valley, Brownsville, TX 78520, USA; 3 School of Medicine, University of Texas Rio Grande Valley, Edinburg, TX 78539, USA

**Keywords:** MASLD, depression, protein–protein interaction, Mexican Americans, gene expression phenotypes

## Abstract

**Background**: Metabolic dysfunction-associated steatotic liver disease (MASLD) and depression frequently occur together. Identifying the genes that influence both MASLD and depression may facilitate the discovery of biological pathways associated with disease risk. **Methods**: We recruited 525 participants from Mexican American families living in the Rio Grande Valley of south Texas. We collected clinical data, biometric measurements, hepatic health assessments using Vibration-Controlled Transient Elastography (VCTE), and depression evaluations determined with the Beck Depression Inventory-II. We estimated the heritability (h^2^) of MASLD-related measures, depression status, aspartate aminotransferase (AST), alanine aminotransferase (ALT), the AST/ALT ratio, and Vibration-Controlled Transient Elastography measurements. For each gene, we derived a genetic endophenotype representing its expression level. We then performed functional network and gene ontology enrichment analyses to characterize the underlying protein pathways. **Results**: We observed significant associations between the expression of two genes, Thyroid Hormone Receptor-Associated Protein 3 (*THRAP3*) (h^2^ = 0.56 [0.45, 0.67]) and ADAM Metallopeptidase with Thrombospondin Type 1 Motif 7 (*ADAMTS7*) (h^2^ = 0.66 [0.55, 0.77]), with depression and multiple MASLD-related phenotypes. We identified 351 genes with expression levels significantly correlated with one or more MASLD phenotypes and depression. Among these, five genes—*ADAMTS7*, *THRAP3*, *CHPM4A*, *RAB9A*, and *PDIA3*—were jointly associated with three phenotypes: AST/ALT, ALT, and Controlled Attenuation Parameter (CAP kPa). Based on the Fisher Combined Test, only *THRAP3* (*p* = 3.0 × 10^−2^) and *ADAMTS7* (*p* = 2 × 10^−2^) were jointly significant for depression (BDI-II) and AST, ALT, AST/ALT ratio, FAST, and CAP (kPa). We present a protein–protein interaction network comprising nodes (proteins) and edges (interactions), and a gene ontology enrichment analysis of cellular components. **Discussion**: Our findings highlight pleiotropic genes underlying MASLD and depression. Two genes, *ADAMTS7* and *THRAP3*, warrant further investigation as potential targets for therapeutic interventions to manage MASLD and depression among Mexican Americans. These results may improve our understanding of the pathways involved in these two diseases, advance current research, and contribute to improvements in personalized medicine. **Conclusion**: We identified possible shared gene expression phenotypes linking MASLD and depression, which may provide insight into a common molecular underpinning. Pathway enrichment and gene analysis were used to help refine networks and enhance our understanding of complex gene-environmental interactions and their implications for precision medicine.

## 1. Background

### 1.1. Metabolic Dysfunction-Associated Steatotic Liver Disease

Metabolic dysfunction-associated steatotic liver disease (MASLD) is a multifactorial complex disorder arising from the interplay between genetic, molecular, and environmental factors [1]. It is the most prevalent cause of chronic liver disease worldwide [1,2]. The precise mechanisms underlying MASLD pathogenesis are incompletely understood, and a unified mechanism of action has yet to be established. MASLD is more prevalent in East Asian Indians, Hispanics, and Asians than in non-Hispanic whites, underscoring potential genetic influences on MASLD development and progression [3,4,5]. Current thought includes a “multiple hit model” which incorporates diet, obesity, inflammation, increased oxidative stress, mitochondrial dysfunction, intestinal dysbiosis, and lipotoxicity [6,7].

Numerous studies have implicated genetic factors in the progression of MASLD, particularly genes involved in inflammation regulation and the immune response [8,9,10]. Genes affecting molecular transport and cell proliferation, differentiation, and apoptosis are potential contributors to MASLD pathogenesis [11,12,13]. Specifically, genes such as *PNPLA3*, *TM6SF2*, *MBOAT7*, *GCKR*, *LIPA*, *HFE*, *HSD17B13*, *MARC1*, *NCAN*, and *FGF21*, and inflammatory pathways (inflammasome-caspase-1 pathway) have been identified as influencing risk for MASLD [4,9,14,15,16]. Pathways involved in the activation of the IL-1 family cytokines. Interferons (IFNs) are implicated in the progression of MASLD to fibrosis and cirrhosis.

### 1.2. Depression

Depression is a prevalent mental illness with a significant genetic component. Depression has been linked to an increased risk for MASLD and other chronic illnesses [17,18,19,20,21,22,23,24]. Risk for depression is determined by both genetic predisposition and environmental factors [25,26,27], with specific genetic risk variants influencing gene expression, inflammatory pathways, and immune response. Evidence supports genetic and environmental risk factors for both depression and MASLD, suggesting possible shared biological pathways between these conditions [28,29,30].

Our earlier research considered depression as an “environment” and identified significant gene—environment (GxE) interactions influencing the risk for hepatic fibrosis in Mexican Americans living in South Texas [28,30,31]. In this report, we aim to explore the role of gene expression, shared associations, protein interactions, and biological pathways that contribute to the pathogenesis of MASLD and depression.

## 2. Materials and Methods

The University of Texas Rio Grande Valley IRB approved the study protocol. All participants provided informed consent before participating in the study.

### 2.1. Participants

The Rio Grande Valley (RGV) is approximately 90% Hispanic, with a high prevalence of diabetes (32%), obesity/overweight (60%), and MASLD (64%) [28]. As described for our ongoing family study [28], we recruited probands who were 18 years of age or older, had a family history of diabetes, had four Mexican-origin grandparents, and had five family members who were eligible to participate [30]. We recruited 525 Mexican American participants (probands and family members) who were evaluated [28]. Information gathered included biometric data (blood pressure, body mass index—BMI), an assessment of depression (Beck Depression Inventory-II-BDI-II), point-of-care HbA1c measures, Vibration-Controlled Transient Elastography (VCTE by FibroScan, Paris, France) measures (including controlled attenuation parameter (CAP) and liver stiffness measurements (LSMs)), and blood samples for future laboratory analyses [28]. More detailed information on the recruitment strategy can be found in earlier publications [28,30,31].

The Beck Depression Inventory-II (BDI-II) was used to assess the degree of depressive symptoms present over the previous two weeks [32,33,34,35]. We followed the standard method of measuring hepatic fibrosis with Vibration Controlled Transient Elastography (a non-invasive diagnostic technique for assessing liver fibrosis and stiffness) [28,36,37]. VCTE quantifies the speed of the shear wave propagated by the ultrasonic wave through the liver. The controlled attenuation parameter (CAP) measures liver ultrasonic attenuation, a marker of steatosis [28,36,37,38]. Fibrosis is reported as the LSM Youden Index in kilopascals (kPa) (Echosens, Paris, France). We measured serum AST and ALT levels (and resultant AST/ALT ratio) using standard biochemical assays. We calculated the FibroScan-AST (FAST) score, which identifies the risk of progressive non-alcoholic steatohepatitis (NASH) (positive predictive value (PPV) of 0·83 (84/101) and a negative predictive value (NPV) of 0·85 (93/110) [36,37,38].

### 2.2. Gene Expression Quantification

We purified total RNA from whole blood collected in PAXgene Blood RNA tubes using the PAXgene Blood 96 RNA Kit (Qiagen, Germantown, MD, USA). Total RNA concentration was estimated using the Picogreen method (Qubit RNA BR kit, Invitrogen, Waltham, MA, USA). RNA quality was estimated using a Qubit RNA Integrity and Quality (IQ) assay (Invitrogen, Waltham, MA, USA) and an RNA Screen tape on the Agilent 4200 Tapestation (Agilent Technologies, Santa Clara, CA, USA). The quality control level was set as samples with an RNA Integrity Number of 7 or higher. RNA samples were processed for Stranded mRNA Sequencing with poly(A) selection (Illumina Stranded mRNA prep, Illumina, San Diego, CA, USA) and sequenced on an Illumina Novaseq 6000 in output mode with a 2 × 50 paired-end read configuration. We set a threshold of 10M paired reads per sample to account for high globin reads. We sequenced sample libraries using Illumina’s HiSeq 2500 (*n* = 467) and NovaSeq (*n* = 288) platforms (Illumina, San Diego, CA, USA). Raw sequencing reads were demultiplexed and stored in compressed FASTQ files using Illumina’s bcl2fastq software (version 2.20.0.422). We estimated Abundance using Kallisto (v0.48.0) [39,40] against the University of California, Santa Cruz (UCSC) hg19 reference transcriptome. No explicit batch correction was applied to account for potential effects arising from the use of two different sequencing instruments. However, samples were randomly distributed across platforms, which mitigates the risk of systematic bias. While this approach may introduce additional background noise, it preserves the integrity of biological signals and avoids overcorrection that could obscure true variation. Estimated transcript counts from Kallisto abundance files were collected into a single matrix. Transcript counts were then normalized by transcript length and across samples using the geTMM method [41,42].

Principal component analysis (PCA) revealed clustering of samples by sequencing instrument, indicating a batch effect. After applying RemoveBatchEffect from the limma package, this batch effect was eliminated, as shown by the absence of instrument-based clustering in the corrected PCA plot (Supplementary Figure S1).

### 2.3. Statistical Genetic Analysis of Liver Disease

We used RNA sequencing to quantify approximately 40,000 transcripts, focusing on 18,000 canonical genes, after excluding microRNAs, long-coding RNAs, and non-specific gene expression measurement data. As in the polygenic model, a phenotype (P) is entirely determined by the sum of an additive genetic component (endophenotype-G) and an environmental component (envophenotype). The endophenotype relates to the genetic component and is directly linked to specific genetic variations, being closer to the biological mechanism than the phenotype or clinical syndrome. Endophenotypes can be utilized in the genetic analysis of complex traits by focusing on more genetically tractable intermediate traits; therefore, they may be linked to pathways from genes to behavior or disease [43].

Members of our group have been involved in the development of approaches to study endophenotypes from a statistical genetic perspective [44]. Our current work represents a continuity of these previous investigations and now uses a novel statistical genetic approach to model endophenotypes, which is the use of the Best Linear Unbiased Prediction (BLUP) algorithm to measure the genetic effects underlying a complex trait rigorously. The virtue of the BLUP approach is that it allows us to remove most, if not all, environmental effects, thereby accentuating the role of genes in the development of complex traits [44,45].

The independent variables in our genome-wide scans, aimed at identifying potential associations with our phenotypes of interest, were endophenotypes corresponding to the original RNA-seq gene expression variables. To determine the significance of endophenotypes that are jointly associated with MASLD and depression, we used Fisher’s combined *p*-value test.

We computed an empirical genetic relatedness matrix using the IBDLD algorithm [45] applied to our WGS data. Cryptic relatedness arises from unknown genetic relatedness in a sample. Given that we work with an empirical genetic relationship matrix rather than a pedigree-based one, all cryptic relatedness has been accounted for.

To test gender differences in the variables, we used the nonparametric Mann–Whitney-Wilcoxon test, which is robust to nonnormality. Each liver-related phenotype (AST, ALT, AST/ALT, CAP, FAST, and kPa) was first regressed on age, sex, age-squared, sex-by-age, and sex-by-age-squared, and residuals were inverse-normal transformed for subsequent analyses. We intentionally did not include BMI or other clinical variables that are highly correlated with liver injury markers as covariates, because statistical genetic theory and simulation work indicate that adjusting for such correlated traits can reduce power to detect genetic effects of interest [46,47]. In this Mexican-American extended-family cohort, the family-based design substantially mitigates confounding by ancestry, so we did not further adjust for ancestry principal components. Data on medications and alcohol intake were incomplete and therefore not included as covariates. The regression residuals derived for each trait were normalized using an inverse normal transformation [48].

We fitted a multivariate linear mixed model in SOLAR in which *ADAMTS7* and THRAP3 transcript levels were evaluated jointly across all MASLD-related liver traits. This analysis provided a phenotype-level joint test of association for each transcript, complementing the primary endophenotype-based scans.

### 2.4. Functional Network and Gene Ontology Enrichment Analysis

The interaction of two or more proteins (protein–protein interaction (PPI)) is crucial in regulating cellular and molecular functions [49]. The STRING database constructs PPI networks by integrating probability from multiple sources of evidence and correcting for the expectancy of random associations [50]. In this study, we constructed the PPI network using STRING database version 12 (https://string-db.org/), exported the TSV file from STRING to Cytoscape, and visualized it using Cytoscape v3.9.1.

Gene Ontology (GO) [51] is a tool for describing protein functions, which may be grouped into three main categories: molecular functions (MF), biological processes (BP), and cellular components (CC). The GO enrichment analysis (cellular component) was performed with *p*-value adjustment to calculate the false discovery rate (FDR) based on the Benjamini–Hochberg approach. Each GO term with an FDR threshold of <0.05 was considered significant.

## 3. Results

### 3.1. Heritability Analysis

We found statistically moderate significant heritabilities for AST (h^2^ = 0.25, *p* < 3.0 × 10^−2^), ALT (h^2^ = 0.41, *p* = 8.1 × 10^−4^), AST/ALT (h^2^ = 0.26; *p* = 4.0 × 10^−3^), CAP (h^2^ = 0.33; *p* = 4.3 × 10^−4^), FAST (h^2^ = 0.36; *p* = 1.5 × 10^−4^), and BDI-II (h^2^ = 0.37; *p* = 7.8 × 10^−6^) as shown in Table 1.

### 3.2. Association of Expression Endophenotypes

Regression diagnostics, including inspection of QQ-plots for liver-related phenotypes, were consistent with adequate model fit after transformation (Supplemental Figure S2). We tested the association between each endophenotype and two sets of phenotypes—MASLD-related phenotypes (AST, ALT, AST/ALT, CAP, FAST) and depression (BDI-II)—using a mixed-methods model to account for the heritable components of the focal phenotypes. We identified 351 genes that were significantly correlated with one or more MASLD phenotypes and depression. Among these, five genes—*ADAMTS7*, *THRAP3*, *CHPM4A*, *RAB9A*, and *PDIA3*—were jointly associated with three phenotypes: AST/ALT, ALT, and CAP. Based on the Fisher Combined Test, only *THRAP3* (*p* < 0.03) and *ADAMTS7* (*p* < 0.02) were jointly significant for depression (BDI-II) and AST, ALT, AST/ALT ratio, FAST, and CAP. CAP and endophenotypes showed statistically significant correlations with ENDO_*ADAMTS7*, which had a weak positive correlation (R^2^ = 0.038, *p* = 0.0014), while ENDO_*THRAP3* exhibited a weak negative correlation (R^2^ = 0.042, *p* = 0.00071).

*ADAMTS7* showed significant association with AST (*p* = 0.05, β = 1.56), ALT (*p* = 4.9 × 10^−4^, β= 4.24), AST/ALT (*p* = 5.2 × 10^−3^, β = −0.05), CAP (*p* = 3.0 × 10^−2^, β = 7.66), FAST (*p* = 1, β = 1.56), and BDI-II (*p* = 2.3 × 10^−3^, β = −0.73 (Table 2).

*THRAP3* showed significant associations with AST (*p* = 5.6 × 10^−2^, β = −1.53), ALT (*p* = 8.8 × 10^−4^, β = −4.1), AST/ALT (*p* = 9.1 × 10^−3^, β= 0.05), CAP (*p* = 2.2 × 10^−2^, β = −8.13), FAST (*p* = 2.2 × 10^−2^, β = −8.13), and BDI-II (*p* = 1.4 × 10^−1^, β = 0.04 (Table 3).

As expected for traits that index liver injury, MASLD-related phenotypes (AST, ALT, CAP, FAST, and kPa) were strongly inter-correlated, whereas correlations with depression (BDI-II) were small.

In a multivariate linear mixed-model sensitivity analysis that jointly considered all MASLD-related liver phenotypes in SOLAR, both *ADAMTS7* and *THRAP3* remained strongly associated with the multivariate liver trait (*ADAMTS7 p* < 7.16 × 10^−8^; *THRAP3 p* < 1.93 × 10^−7^). These results support the robustness of the key signals when accounting for the shared correlation structure among liver phenotypes

### 3.3. Overview of PPI Network and GO Enrichment Analysis for Cellular Component

**Figure 1:**
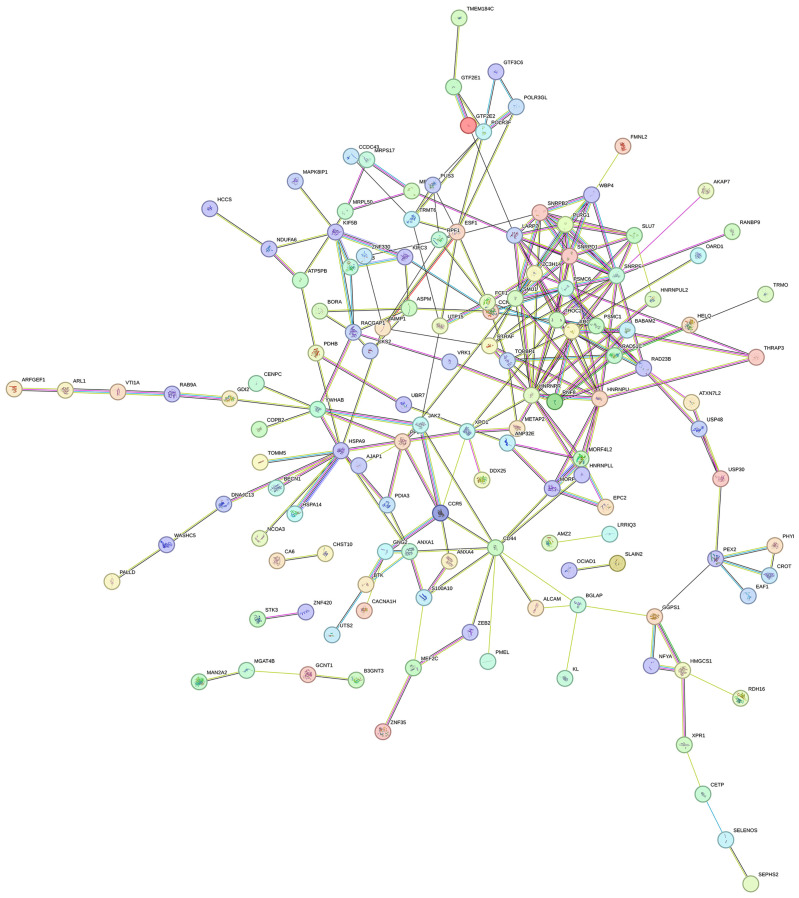
A PPI network comprises nodes and edges, with nodes denoting proteins and edges denoting interactions. Edge thickness reflects the confidence level of the interaction, and node centrality determines node size.

Figure 1 illustrates a network of nodes and edges, where nodes represent proteins, and edges represent protein interactions. The colored nodes indicate the query protein and its first-shell interactors. Several protein clusters are found within the strongly connected PPI network, which is known to be involved in diverse biological activities, including transcriptional control, cell cycle progression, and RNA processing. THRAP3 was identified as a key node presenting protein-protein interactions with HNRNPU, BABAM2, and PSMC6. In addition to THRAP3, other interactions play important roles in metabolism, RNA splicing, stabilization, and the response to DNA damage. THRAP3 is highly connected, suggesting a critical role in transcriptional and metabolic coordination, potential regulatory functions, and an association with pathological manifestations, such as MASLD.

**Figure 2:**
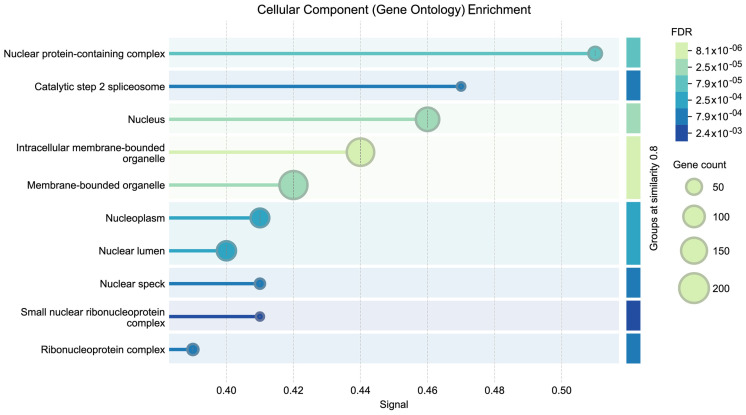
GO enrichment analysis for cellular components (y-axis, specific GO terms; x-axis significance (FDR). Each bubble represents the number of genes associated with a term, and color gradient represents the FDR values, lighter colors indicate more significant terms.

GO enrichment analysis of cellular components is shown in Figure 2, indicating the most significant enriched terms [51]. Using FDR as a measure of significance, the horizontal axis shows the association’s significance level, while the vertical axis shows the GO term associated with the cellular component. Components with the lowest FDR are highly enriched. FDR values are displayed in a gradient of colors, with darker colors denoting terms with less significance and lighter colors denoting terms with greater significance. Bubble size indicates how many genes are associated with each GO term, with larger bubbles indicating more genes. Furthermore, enriched cellular components such as intracellular membrane-bounded organelles or the nucleus provide evidence of their functional importance STRING/GO-CC to process-level enrichment (GO-BP, Reactome, KEGG) with FDR control).

## 4. Discussion

*ADAMTS7* and *THRAP3* share an association with both depression and MASLD, and they may be partially responsible for the interplay between depression and MASLD. *ADAMTS7* and *THRAP3* are negatively correlated, reflecting their opposing effects on inflammation. *ADAMTS7* highly correlates with each endophenotype we measured and shows a notable role in promoting inflammatory processes. *THRAP3* also correlates with each measured endophenotype and suppresses inflammation. Evidence is mounting that many chronic illnesses are related to inflammation pathways.

A Disintegrin and Metalloproteinase with Thrombospondin Motifs 7 (ADAMTS7), a member of the ADAMTS metalloproteinase family, is involved in post-translational modifications by cleaving extracellular matrix proteins including thrombospondin-5, aggrecan, and cartilage oligomeric matrix protein [52,53]. ADAMTS7 expression is upregulated in inflammatory conditions and contributes to tissue remodeling and vascular pathology. Through its effects on extracellular matrix degradation and inflammatory signaling pathways, ADAMTS7 may indirectly influence NLRP3 inflammasome activation and contribute to the inflammatory cascade [52]

Thyroid hormones play a significant role in liver and brain metabolism. The thyroid hormone involves mitosis, gluconeogenesis, cholesterol conversion to bile acids, apolipoprotein A1, and the modulation of HDL receptors. The thyroid hormone is involved in the brain’s development, function, and myelination. An example of its importance in brain development is congenital hypothyroidism, one of the most preventable causes of intellectual disability [54]. Thyroid receptors function as transcriptional factors through interaction with co-activators, co-repressors, and thyroid hormone response elements located in the regulatory regions of the thyroid hormone gene.

Thyroid hormone receptor-associated protein 3 (THRAP3) plays a role in transcriptional regulation and DNA repair [55]. Chronic inflammation, oxidative stress, and metabolic dysregulation contribute to DNA damage. In response to DNA damage, THRAP3 is strongly phosphorylated [56,57] and directly interacts with and upregulates PPAR-γ activity [58,59]. PPAR-γ is a nuclear receptor protein that mediates an anti-inflammatory response by inactivating the protein complex Nuclear Factor (NF)-kappa B/Rel family of transcription factors (NF-kB) [59]. Therefore, depletion or inactivation of THRAP3 causes unregulated activity of NF-kB and subsequent inflammation responses. This ultimately leads to cells becoming hypersensitive to DNA-damaging agents [55] because the inflammation/immune response is not inhibited.

In differentiated adipocytes, Thrap3 regulates PPARγ transcription and acts as a master regulator of adipocyte differentiation and lipid storage. The protective role of THRAP3 involves reducing inflammation [60]. This intricate interplay between THRAP3, PPAR-γ, and NF-κB has significant implications for various diseases, particularly chronic inflammation and metabolic dysregulation, such as Metabolic dysfunction-associated steatotic liver disease (MASLD) [60].

THRAP3 is a critical regulator of transcriptional control, DNA repair, and inflammatory responses. Its depletion or dysfunction can lead to a pro-inflammatory state and increased susceptibility to DNA damage, potentially contributing to the development and progression of various diseases [61]. Understanding these complex interactions provides valuable insights for developing targeted therapeutic approaches for chronic inflammation and DNA damage [57,60].

### 4.1. Recent Advances in MASLD Treatment

**Figure 3:**
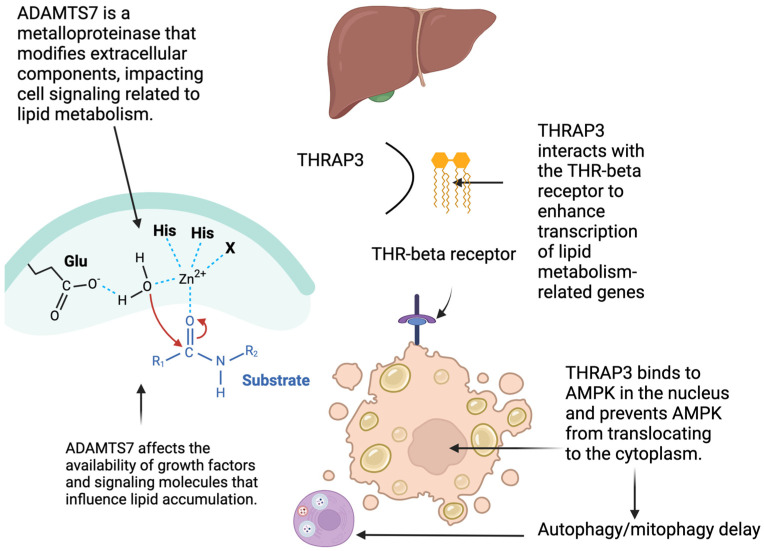
The Role of THRAP3 and ADAMTS7 in the Evolution of MASLD. Image created by biorender.com—Manusov.

Figure 3 presents a mechanistic model that emphasizes the function of THR-β signaling and integrates the roles of THRAP3 and ADAMTS7 in MASLD evolution. Activation of THR-β (thyroid hormone receptor-β) in hepatocytes stimulates lipid metabolism by promoting beta-oxidation, increasing mitochondrial biogenesis, and enhancing the clearance of intracellular triglycerides. These processes lower inflammation and reduce hepatic steatosis. Critical for reducing the progression to fibrosis. THRAP3, identified in our study as associated with both MASLD and depression, has been shown to regulate transcriptional programs downstream of nuclear receptors, such as THR-β, and to interact with pathways controlling inflammation and metabolic function. Thus, THR-β activation may exert beneficial hepatic effects by stimulating lipid catabolism and by modulating THRAP3′s role in transcriptional regulation and inflammatory signaling.

The figure illustrates that optimal THR-β function, possibly in tandem with THRAP3, can antagonize profibrotic influences such as those mediated by ADAMTS7, thereby attenuating extracellular matrix remodeling and reducing the risk of progression to advanced liver disease. This expanded mechanistic insight highlights the relevance of THR-β–THRAP3 interactions in linking gene expression networks to metabolic and fibrotic outcomes in MASLD.

### 4.2. Metabolic Dysfunction-Associated Steatotic Liver Disease and Depression

ADAMTS7 is involved in multiple areas of inflammation that lead to fibrosis, including coronary artery disease, osteoarthritis, and liver disease. [61,62,63,64,65] ADAMTS7 expression is regulated by inflammatory stimuli. The mRNA and protein levels of ADAMTS7 are induced by pro-inflammatory cytokines, including tumor necrosis factor-alpha and interleukin 1 beta [66] The anti-inflammatory cytokine transforming growth factor-beta down-regulates ADAMTS7 expression Hepatic progenitor/oval cells (OC) can differentiate into hepatic parenchymal cells and hepatocytes during regeneration [66]. Matrix remodeling and metalloprotease activities are associated with hepatocyte proliferation, and when inhibited, the fibrogenic response leads to liver damage [67].

ADAMTS7 has been shown to regulate hepatic lipid metabolism, suggesting another potential role in metabolic liver diseases. A microRNA, miR-29a/b, negatively regulates ADAMTS7 expression [66,67] and plays an essential role in liver fibrosis characterized by Extracellular Matrix Proteins (ECM), such as collagens, in the liver. miR-29a/b expression is decreased in patients with liver fibrosis and is thought to contribute to the upregulation of ECM genes and sub6equent accumulation of ECM proteins in the liver [68]. There are pro-inflammatory element binding sites, including NF-kB and AP-1 sites, within the ADAMTS7 promoter [68]. NF-kB and signal transduction networks are associated with metabolic syndrome, type 2 diabetes, obesity, and coronary artery disease. The ECM is one of the most important regulators in the hepatic progenitor cells (HPCs) that provide a physical scaffold and support for hepatic cells [69,70].

Depression is associated with the dysregulation of the immune system [71]. A proposed mechanism linking MASLD and depression with ADAMTS7 is through the upregulation of ADAMTS7 and increased inflammasome activity [72,73]. The result is increased IL-6. IL-6 effects are targeted towards depression through activation of indoleamine-2,3-dioxygenase (IDO), which catalyzes tryptophan degradation via the kynurenine pathway, leading to decreased central serotonin availability and contributing to depressive symptoms [74]. ADAMTS7 expression is increased in the brains of mice exposed to chronic social stress, and genetic variations in the ADAMTS7 gene may be associated with an increased risk for depression in humans. Exposure to childhood trauma is associated with higher levels of ADAMTS7 expression in patients with major depressive disorders [75].

THRAP3 may be involved in depression through the AMPK-mediated hippocampal autophagic response, the NLRP3 inflammasome, and AMPK-mediated neurotrophin secretion [76]. THRAP3 and ADAMTS interact with inflammatory pathways, supporting the inflammation theory of both MASLD and depression [75,76,77,78].

### 4.3. Summary

Gene expression occurs throughout an individual’s lifetime and is associated with genomic plasticity. Up-regulation of Thrap3 is protective, whereas up-regulation of ADAMTS7 leads to increased fibrosis. ADAMTS7 and THRAP3 are regulatory elements that influence the NF-kappaB inflammation signaling network.

Our results demonstrate that ADAMTS7 and THRAP3 are both associated with MASLD and depression. ADAMTS7 is involved in multiple canonical pathways regulating cell proliferation, differentiation, migration, and extracellular matrix (ECM) remodeling. Both ADAMTS7 and THRAP3 participate in NF-kB signaling pathways, which have been implicated in both depression and liver disease. The joint association of ADAMTS7 and THRAP3 transcripts at the intersection of liver fibrosis and depression further supports the inflammatory theory of depression. It illustrates how genes interact with inflammatory signaling to accelerate fibrosis in MASLD.

Researchers are focusing on ADAMTS7 in the treatment of other inflammatory diseases. THRAP3 depletion has been shown to increase tumor necrosis factor-alpha–induced lipolysis, pro-inflammatory gene expression, and activation of signal-regulated kinases. Our findings support that these proteins may be targeted to reduce the progression of MASLD, especially in Mexican Americans with depression.

Perhaps ADAMTS7 and THRAP3 will improve prediction models for depression and MASLD, serve as therapeutic targets, or enhance our understanding of the role of inflammation in chronic disease. Although ADAMTS7 is associated with MASLD and depression, the protein was not included in the pathways we analyzed. This relationship has not yet been added to the database, making our findings important for documenting gene interactions.

### 4.4. Limitations

The study design was cross-sectional, relying on data from a single time point. This limited our ability to establish causal relationships or determine the directionality of the observed associations. Although statistically significant associations were identified, the observed R^2^ values (0.038–0.042) indicate that genetic markers explain only a small fraction of the variance in liver and depression. The weak correlations reported should be interpreted with caution. Given the high inter-correlations among liver phenotypes, individual regression coefficients should be interpreted cautiously, as small differences in model specification can shift estimates among correlated outcomes even when overall patterns remain consistent.

In addition, further investigation is necessary to identify and characterize other environmental factors, including potential confounders, such as diet, medication use, comorbidities, socio-economic factors, and genes that contribute to these interactions. Future studies should aim to elucidate the functional mechanisms by which ADAMTS7 and THRAP3 influence the development and progression of MASLD in the context of depression. We also acknowledge limitations in covariate adjustment. We deliberately avoided including BMI and other liver-related clinical variables as covariates because of their strong correlation with the MASLD phenotypes, which can reduce power to detect genetic effects. Our predominantly Mexican-American, extended-family design helps control for population structure without explicit ancestry PCs, but we lacked complete and harmonized data on medications, alcohol intake, and other lifestyle factors, and were therefore unable to model them directly. These factors could still contribute to residual confounding and should be addressed in future studies with richer exposure data.

We acknowledge that the effect sizes of the identified genetic variants are small, making it challenging to establish robust direct connections to disease outcomes. Importantly, such modest effect sizes of genetic variants in relation to complex disease are, however, predicated on a network dyshomeostasis model [79,80]. According to this model of network causation, numerous genes with small effects act in concert with multiple environmental factors over time to cumulatively cause complex diseases. Future studies should aim to elucidate the functional mechanisms by which ADAMTS7 and THRAP3 influence the development and progression of MASLD in the context of depression.

## 5. Conclusions

Our study uses gene expression data to demonstrate molecular function and provides valuable insights into MASLD and depression among Mexican Americans. We found that THRAP3 and ADAMTS7 are both associated with depression and MASLD and warrant investigational focus as potential targets for therapeutic intervention.

While our study demonstrates joint associations between ADAMTS7 and THRAP3 expression and MASLD-related traits and depression, these findings are preliminary and lack support from external genetic linkage analyses, including colocalization with MASLD and MDD GWAS loci, cross-trait linkage disequilibrium score regression, or Mendelian randomization. Our results should be viewed as hypothesis-generating rather than as evidence for therapeutic targets or prediction markers. Further research utilizing integrative genomics approaches is necessary to establish whether ADAMTS7 and THRAP3 are causally implicated formally and to clarify their specific roles within shared biological pathways across disease contexts.

## Figures and Tables

**Table 1:** Sample size differences reflect missing VCTE data.

Trait	Heritability h^2^	Standard Error	Sample Size	*p*-Value
AST	0.25	0.14	525	3.1 × 10^−2^
AST	0.41	0.13	525	8.1 × 10^−4^
AST/ALT	0.26	0.10	525	4.0 × 10^−3^
CAP	0.33	0.10	525	4.3 × 10^−4^
FAST	0.36	0.12	475	1.5 × 10^−2^
BDIII	0.37	0.10	525	7.8 × 10^−6^

**Table 2:** Association of *ADAMTS7* with measures of depression and liver health.

Trait	Beta (β) *	SE	*p*-Value	q-Value
AST	1.56	0.80	0.05	1
ALT	4.24	1.21	4.9 × 10^−4^	0.35
AST/ALT	−0.05	0.02	5.2 × 10^−3^	0.99
CAP	7.66	3.64	3.0 × 10^−2^	0.81
FAST	1.56	0.80	1	1
BDI-II	−0.73	0.24	2.3 × 10^−3^	0.10

* β corresponds to the regression coefficient of each phenotype as an endophenotype.

**Table 3:** Association of *THRAP3* with measures of depression and liver health.

Trait	Beta (β) *	SE	*p*-Value	q-Value
AST	−1.53	0.80	5.6 × 10^−2^	1
ALT	−4.06	1.21	8.8 × 10^−4^	0.35
AST/ALT	0.05	0.02	9.1 × 10^−3^	0.99
CAP	−8.13	3.52	2.2 × 10^−2^	0.77
FAST	−0.01	0.01	5.6 × 10^−2^	0.21
BDI-II	0.04	0.24	1.4 × 10^−1^	0/09

* β corresponds to the regression coefficient of each phenotype as an endophenotype.

## Data Availability

The data presented in this study are available on request from the corresponding author. The data are not publicly available due to privacy or ethical restrictions.

## Supplementary Materials

The following supporting information can be downloaded at: https://www.mdpi.com/article/10.3390/genes17030343/s1, Figure S1. Corrected PCA of RNA Abundance. Figure S2. Q-Q Plots of Hepatic Traits.
