# Supplementary figures and images for: Genetic Variants and Molecular Components Associated with Metabolic Dysfunctional-Associated Steatotic Liver Disease and Depression: Shared Association of ADAMTS7 and THRAP3
Source: Genes (Basel). 2026 Mar 19;17(3):343. doi: 10.3390/genes17030343 (PMC13026727; doi:10.3390/genes17030343)

ALT

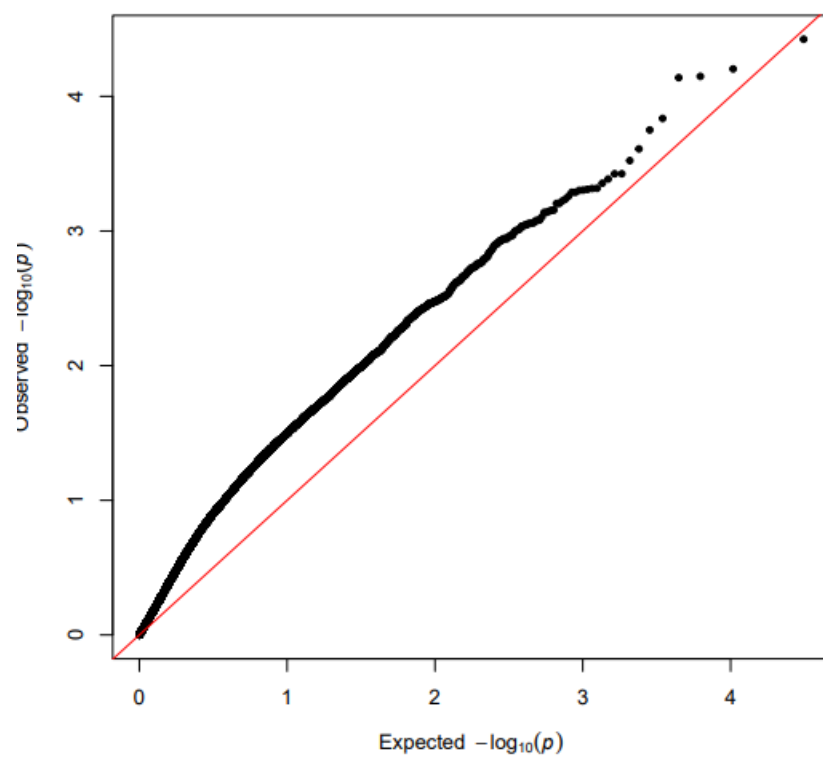

AST

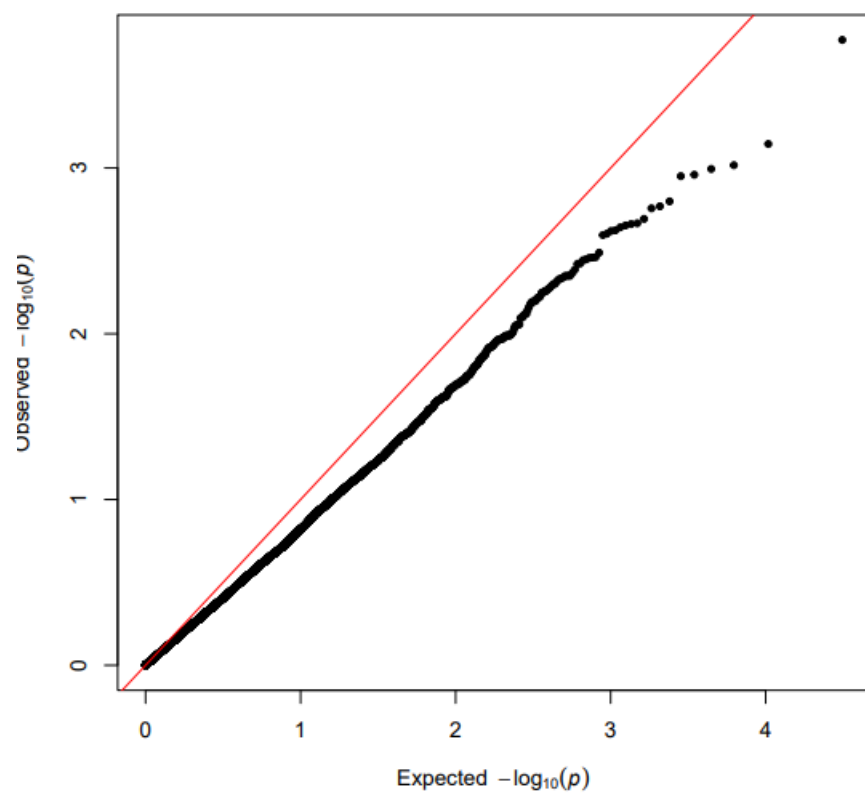

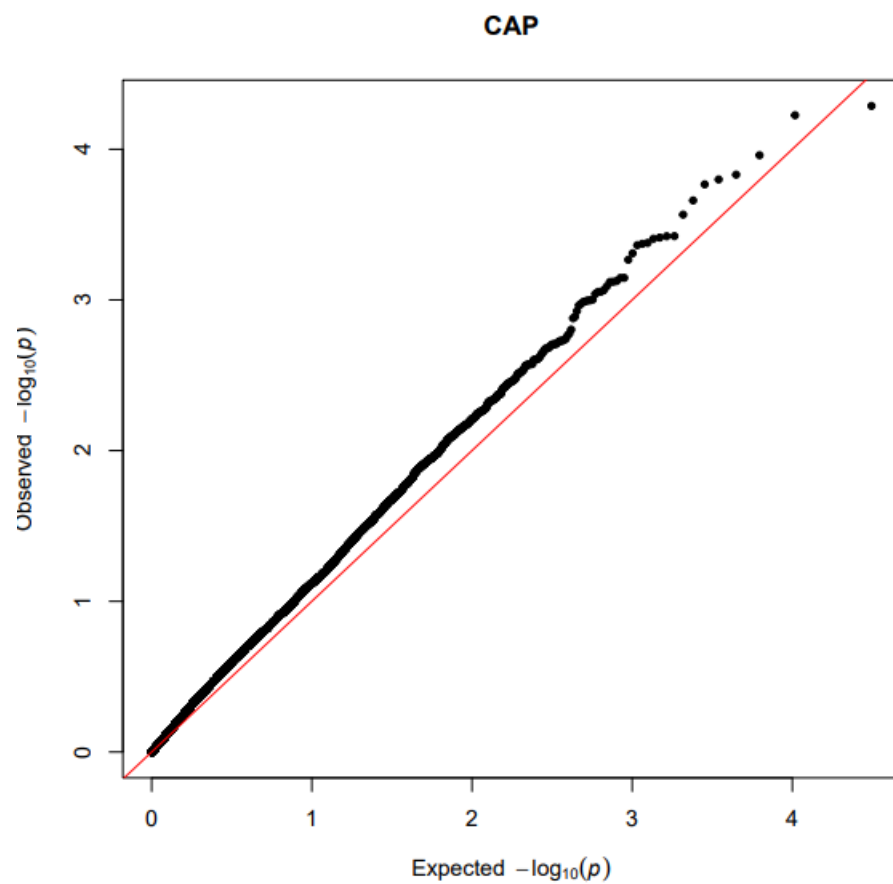

Supplementary figure 2. QQ-plots of Hepatic Traits

Supplement: Supplementary file 1 [file genes-17-00343-s001.zip › genes-3887640-supplementary.pdf]
